# Bimanual grasping does not adhere to Weber’s law

**DOI:** 10.1038/s41598-017-06799-4

**Published:** 2017-07-25

**Authors:** Tzvi Ganel, Gal Namdar, Avigail Mirsky

**Affiliations:** Department of Psychology, Ben-Gurion University of the Negev, Beer-Sheva, 8410500 Israel

## Abstract

According to Weber’s law, a fundamental principle of perception, visual resolution decreases in a linear fashion with an increase in object size. Previous studies have shown, however, that unlike for perception, grasping does not adhere to Weber’s law. Yet, this research was limited by the fact that perception and grasping were examined for a restricted range of stimulus sizes bounded by the maximum fingers span. The purpose of the current study was to test the generality of the dissociation between perception and action across a different type of visuomotor task, that of bimanual grasping. Bimanual grasping also allows to effectively measure visual resolution during perception and action across a wide range of stimulus sizes compared to unimanual grasps. Participants grasped or estimated the sizes of large objects using both their hands. The results showed that bimanual grasps violated Weber’s law throughout the entire movement trajectory. In contrast, Just Noticeable Differences (JNDs) for perceptual estimations of the objects increased linearly with size, in agreement with Weber’s law. The findings suggest that visuomotor control, across different types of actions and for a large range of size, is based on absolute rather than on relative representation of object size.

## Introduction

According to Goodale and Milner’s account of the organization of the primate visual system, the dorsal ‘action’ pathway, which includes regions within the parietal lobe, enables a flexible control of actions directed to objects in the environment, whereas the ventral ‘perception’ pathway provides a rich and detailed representation of the perceptual scene^1–3^. This suggestion of a functional separation between the two visual systems underlying action and perception has been supported by converging evidence obtained from different domains^4^. Behavioral findings indicate that visuomotor interactions with objects are not always governed by well-established psychophysical principles of visual perception^5^. It has been argued that unlike visual perception that computes object size in a relative manner, visuomotor control is subserved by an analytic processing style^6^. Startlingly, Weber’s law, psychology’s first and still most widely known principle of relative perception, does not apply when grasping objects^7, 8^.

According to Weber’s law, perceptual resolution is relative rather than absolute. Weber’s law was found to hold for most aspects of human perceptual experience, with only few violations at the extreme ends of the perceptual magnitude spectrum^9^. In the case of the perception of object size, visual resolution can be regarded as relative because it depends on the size of the target object. Specifically, according to Weber’s law, perceptual resolution decreases in a linear fashion with an increase in size. Wealth of previous research conducted in our lab and in other labs have shown, however, that unlike perceptual estimations that obey Weber’s law, the resolution of grasping movements during unimanual grasps is not affected by object size and therefore violates Weber’s law^7, 8, 10–14^. It has been argued therefore, that visuomotor control is not governed by the same psychophysical laws that govern perception.

We note however, that the dissociation between perception and action in terms of their adherence to Weber’s law was examined only for single-handed, *unimanual*, precision grasps which are limited by the range of stimulus sizes available for grasp. After all, due to obvious biomechanical constrains of the maximum opening between the fingers, unimanual grasps can be directed to a restricted range of object sizes^15^. Indeed, previous studies have typically looked at the influence of Weber’s law for objects ranging between 20–70 mm in size, which in turn represent a “comfort range” for unimanual grasps. It has been noted that objects beyond this range may produce a ceiling effect in grip aperture due to biomechanical constrains^15–17^. The potential influence of such ceiling effects can be even more dramatic when grasps are performed under abnormal, challenging visual conditions, such as when they are performed without visual feedback of the hand or of the target object. In such situations, participants tend to increase their “safety margin” of grip aperture prior to grasp, which in turn limits the size of their comfort grasp range^16^. Due to the fact that JNDs are measured by the within-subject variability (STDV) of grip apertures, ceiling effects in such atypical situations may sometimes lead to reduction in variability with object size when very large objects are used as targets, or when finger apertures are considerably large.

It is not surprising therefore, that most previous studies used objects within the comfort size range to avoid potential ceiling effects. At the same time, however, the usage of a restricted range of stimulus sizes potentially limits the generality of the observed dissociation between perception and action in terms of their adherence to Weber’s law. The purpose of the current study was to test the effect of Weber’s law on visuomotor control (and on perceptual estimations) across a different task and across a considerably larger stimulus size range. To this purpose, we asked participants to grasp or to estimate the sizes of large objects using both their hands. Bimanual grasping has been proposed to rely on overlapping, although partially distinct neural mechanisms than those that mediate the control of unimanual, single-hand grasping^18^. Importantly, JNDs could be measured for bimanual grasping across a considerably larger range of objects sizes compared to unimanual grasping, without the hazard of potential biomechanical constrains.

In the current study we compared the adherence of grasping and the adherence of perceptual estimations to Weber’s law for bimanual grasping and estimation tasks. That also allowed testing for possible dissociations between perception and action for objects that are considerably larger in size compared to those used in previous studies. Participants were asked to grasp (or to perceptually estimate the size of) large objects (150–450 mm) using both their hands. Unlike for unimanual grasps, bimanual grasps are not limited by biomechanical limitations within that size range. We hypothesized that similarly to unimanual grasps, bimanual grasps would escape the influence of Weber’s law, whereas perceptual estimations of the size of the same objects would at the same time adhere to Weber’s law.

## Materials and Methods

### Participants

A group of 16 right-handed students (9 females, mean age: 24.1, stdev: 1.81) from Ben-Gurion University of the Negev participated in the main experiment (8 participants in each experimental condition). A group of 8 different participants participated in the control, open-loop grasping experiment (1 female, mean age: 25.7, stdev: 2.81). An additional group of 8 participants participated in the manual estimation control experiment for which haptic feedback was provided (3 females, mean age: 23.9, stdev: 3.22). Participants received an equivalent amount of 8$ for their participation. The experimental protocol was approved by the ethics committee of the Department of Psychology in Ben-Gurion University of the Negev. The study adhered to the ethical standards of the Declaration of Helsinki.

All participants signed a consent form prior to their participation in the experiment. The manuscript contains no information or images that could lead to identification of a study participant.

### Design and procedure

**Figure 1:**
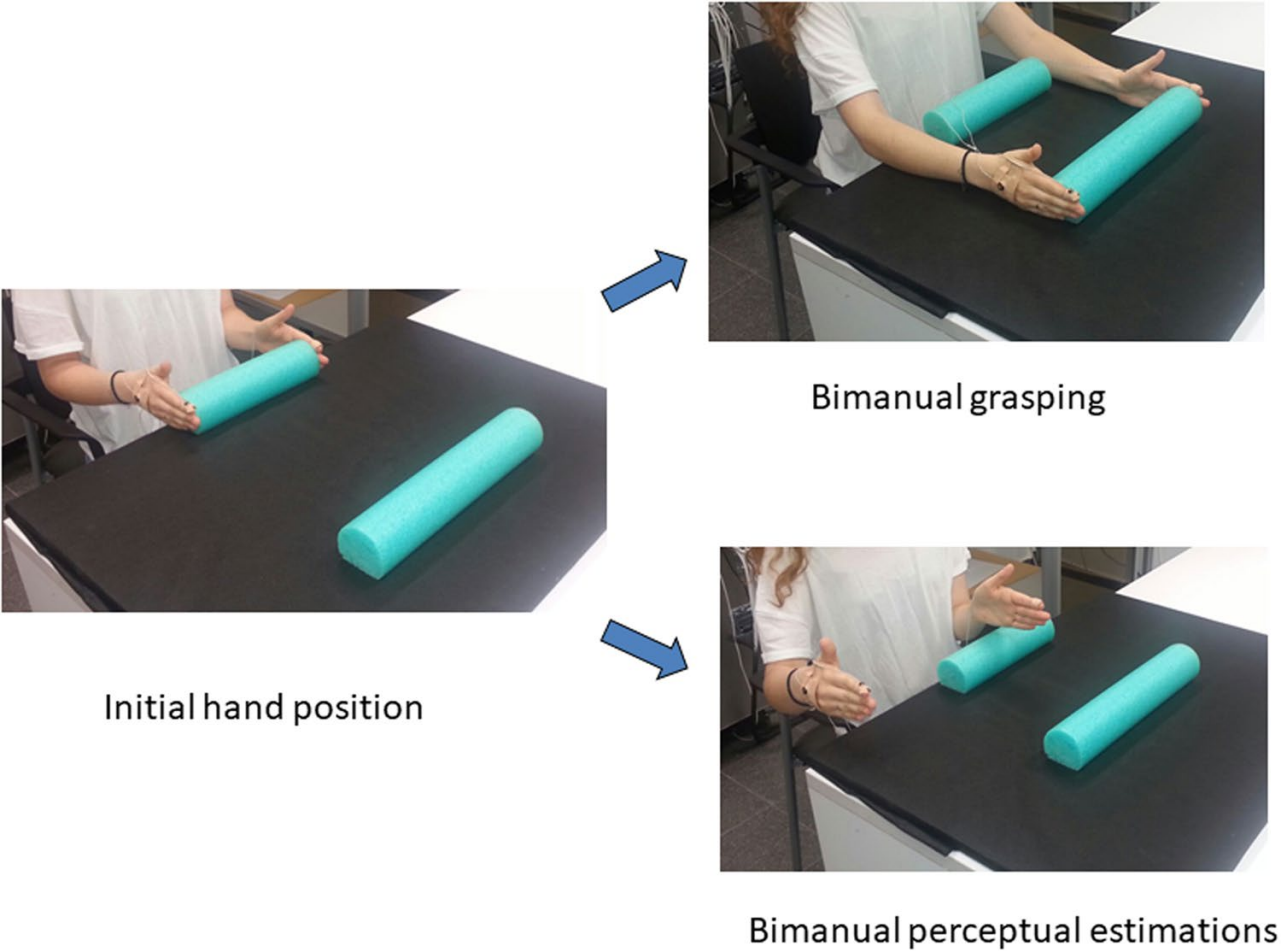
An illustration of the experimental design. Participants were asked to either grasp each object with both their hands (grasping condition) or to estimate its size using both their hands (perceptual estimations condition). To prevent a possible confound of hand velocity, the initial opening between the hands prior to grasping was always set to be 100 cm smaller than the size of target object^10^.

Due to the large differences between the sizes of the objects and to prevent unwarranted effects of hand velocity on early movement trajectories, the initial distance between the two hands was set to be perfectly correlated with the target object size^10^. This allowed computing JNDs throughout the entire movement trajectory free of potential confounds of aperture velocity. The initial distance was set to be always 100 mm smaller than the size of target object (see Fig. 1).

4 objects of different sizes (150, 250, 350, and 450 mm in length) made from polystyrene were presented in each condition (15 repetitions of each object) in a pseudo-random fashion. The starting position and the target objects were aligned with the participants’ body midline. Participants were asked to either pick each object up with their hands (bimanual grasping condition) or to estimate the length  of each object (perceptual estimations condition). Participants were asked to perform both tasks accurately and in a natural manner.

The main experiment consisted of two separate, closed-loop conditions, a bimanual grasping task and a bimanual estimation task. In the grasping task, the participants were asked to grasp each object along its length using both their hands, and then to lift the object a few centimeters in the air before placing it back on the tabletop and returning their hands to the starting position. The starting position was composed of an object which was always 100 cm smaller in length than the target object, positioned 40 cm in front of the participant. In the manual estimation condition, the participants were asked to estimate the object’s length using both their hands. They performed their estimations about 20 cm to the right of the initial starting position by opening their hands to an extent that matched the perceived length of the object. This was done to ensure that participants would estimate the object’s size and would not use other strategies such as aligning their hands with the edges of the target object. LCD liquid-crystal glasses were used to control exposure time. The opening of the goggles in each trial served as a “go” cue to initiate grasping or estimations movements. Vision was allowed throughout the entire movement in both experimental conditions. The first 4 trials in each condition were considered as practice trials and were not included in the analysis.

### Kinematic recordings and data analysis

An Optotrak Certus device (Northern Digital, Waterloo, ON), was used to measure position data and to track hand trajectories. Infrared light-emitting diodes were placed on the top of the distal phalanges of the right and left index fingers. Movement trajectory was collected at a 200-Hz sampling rate from both diodes. Movement onset was determined as the point in time at which the aperture between the fingers increased by more than 0.2 mm for at least ten successive frames (50 ms). Movement offset was determined as the point in time at which the aperture between the fingers changed by no more than 0.2 mm for at least twenty successive frames (but only after reaching the MGA in the grasping condition). Trials were excluded from the analysis if the infrared diodes placed on the fingers were not visible to the camera while moving toward the object or if the participant failed to grasp the object properly and dropped it while trying to lift it off the table. This resulted in 9% and 8% of the trials excluded from the analysis in the grasping and the manual estimation conditions, respectively, due to camera visibility and less than 1% of the trials excluded due to lift errors. In the two control experiments, 4% of the trials were excluded due to camera visibility and 1% due to lift errors.

The experimental design in which a perfect correlation was induced between initial finger opening and target object size allows an effective exploration of at the adherence to Weber’s law during grasping and perceptual estimations across the entire movement trajectory^8, 10^. In order to calculate JNDs during the movement trajectory, we divided each trial’s trajectory into 11 intervals equal in length (0–100%). We have also derived the Maximum Grip Aperture (MGA) for each trial. In the manual estimation (perception) condition, we measured the aperture between the fingers at the end of the estimation trial. The JNDs were calculated using the method of adjustment^10^. In particular, JNDs were calculated by the respective within-subject standard deviations of the responses for each stimulus size.

### Data Availability

The datasets generated during and/or analyzed during the current study are available from the corresponding author on reasonable request.

## Results and Discussion

### Grip apertures

**Figure 2:**
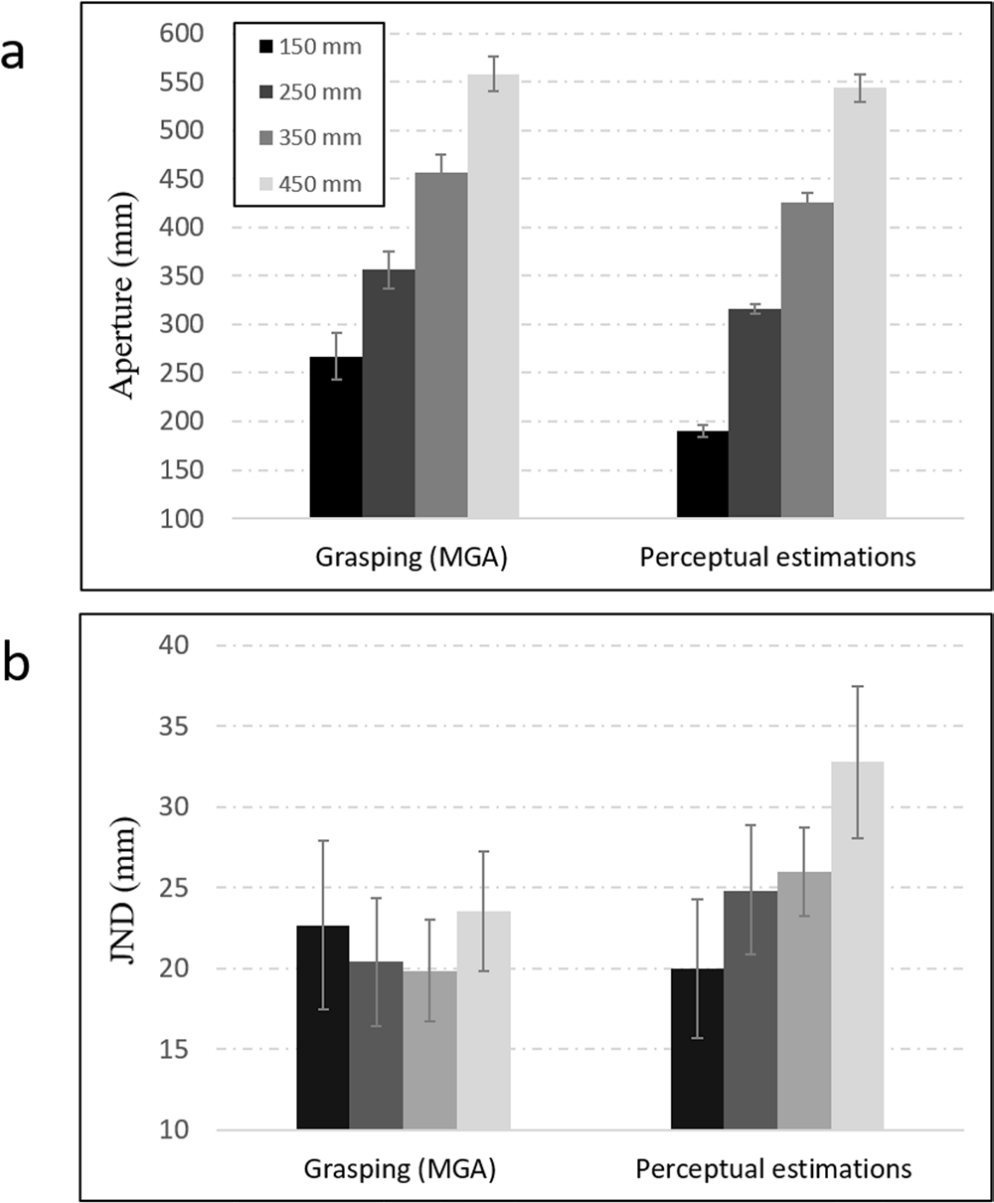
Average grip apertures (**a**) and JNDs (**b**) at the point of maximum grip aperture (MGA) for grasping and for perceptual estimations. Unlike perceptual estimations that adhered to Weber’s law, grasping trajectories did not increase with object size and were therefore immune to Weber’s law. Error bars represent standard errors of the mean.

Average grip apertures for bimanual grasping (during MGAs) and for bimanual perceptual estimations are presented in Fig. 2a. As can be seen in the figure, apertures increased with an increase in object size for both grasping and perceptual estimations. A mixed-design ANOVA with task (grasping/manual estimations) and object size revealed a main effect of size (F(3,42) = 914.2, p < 0.001, η^2^_p_ = 0.98). The main effect of experimental condition was not significant (F(1,24) = 3.98, p < 0.05, η^2^_p_ = 0.22). A significant interaction between condition and size was also found (F(3,42) = 8.3, p < 0.001, η^2^_p_ = 0.37). This interaction stemmed from the difference in the linear slopes (in relation to the real physical sizes of the objects) that characterized bimanual estimations (average slope = 0.87) and bimanual grasping (average slope = 1.03)^19^.

### JNDs

Importantly, marked differences were found in the correspondence between JNDs and object size for bimanual grasping and for bimanual perceptual estimations. As can be seen in Fig. 2b, JNDs for grasping during the point of MGA did not increase with object size (linear component: F(1,14) <1), in violation of Weber’s law. Unlike for bimanual grasps, JNDs for bimanual perceptual estimations increased in a linear fashion with object size (linear component: F(1,14) = 16.2, p < 0.001, η^2^_p_ = 0.54), in adherence to Weber’s law. A significant interaction between the linear components of bimanual grasping and perceptual estimations indicated that the two tasks were statistically different in terms of the effect of size on JNDs  (F(1,14) = 7.25, p < 0.05, η^2^_p_ = 0.34).

### JNDs during movement trajectory

**Figure 3:**
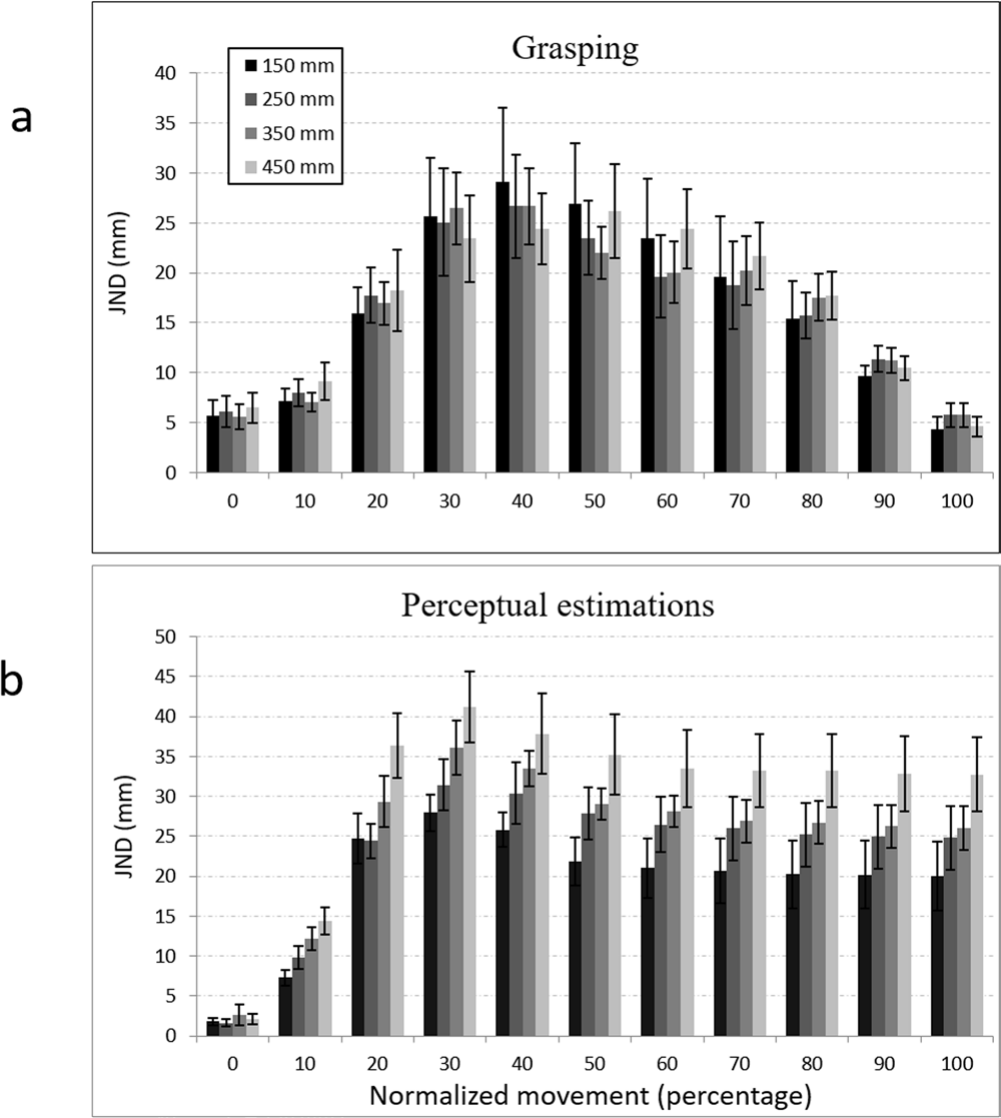
JNDs at different stages of the normalized movement during grasping (**a**) and during perceptual estimations (**b**). Perceptual estimations adhered to Weber’s law throughout the entire movement trajectory. In sharp contrast, grasping trajectories were immune to Weber’s law throughout the entire movement trajectory. Error bars represent standard errors of the mean.

Several previous studies have looked at the correspondence between JND and object size during single-hand grasping throughout the entire movement trajectory^10, 11, 13, 20^. To explore this relationship in bimanual grasping and perceptual estimations, we computed JNDs during different stages of the movements in both tasks. As can be seen in Fig. 3, the dissociation observed between grasping and perception during the point in which MGA was achieved was also observed throughout the entire movement trajectory.

A mixed ANOVA design of task (grasping/estimations), object size, and movement (11 segments, see methods) revealed main effects of movement (F(10,140) = 35.2, p < 0.001, η^2^_p_ = 0.72), size (F(3,42) = 6.74, p < 0.001, η^2^_p_ = 0.32), and task (1,14) = 5.46, p < 0.05, η^2^_p_ = 0.28), which resulted from larger JNDs in the grasping compared to the estimations condition. Most importantly, a task X size interaction indicated that JNDs were differently affected by object size for grasping compared to perceptual estimations (F(3,42) = 5.79, p < 0.01, η^2^_p_ = 0.29). Specific comparisons revealed that JNDs during grasping were not affected by object size, in violation of Weber’s law (linear component: F(1,14) <1). JNDs during bimanual perceptual estimations, however, linearly increased with size (linear component: F(1,14) = 36.42, p < 0.001, η^2^_p_ = 0.28), obeying to Weber’s law. Specific comparisons of the linear component of size in each segment of the movement reinforced the argument that the dissociation between perception and action was observed throughout the entire movement; no effects of size were found in any part of the movement during grasping. In contrast, significant effects of size were found during each segment (10–100%) of the movement during perceptual estimations. Significant interactions were also found between task and movement (F(10,140) = 5.82, p < 0.001, η^2^_p_ = 0.29), and task, movement and size (F(30,420) = 1.51, p < 0.05, η^2^_p_ = 0.09).

Analysis of the reaction times to initiate the movement did not reveal a significant relationship between RT and objects size for both the bimanual grasping condition (271, 303, 293, and 292 ms for the 150, 250, 350, and 450 mm objects respectively, F(1,7) = 1.66, p > 0.05) and the bimanual perceptual estimations condition (488, 515, 595, and 599 ms for the 150, 250, 350, and 450 mm objects respectively, F(1,7) = 2.23, p > 0.05).

### Controlling for online visual feedback during grasping

**Figure 4:**
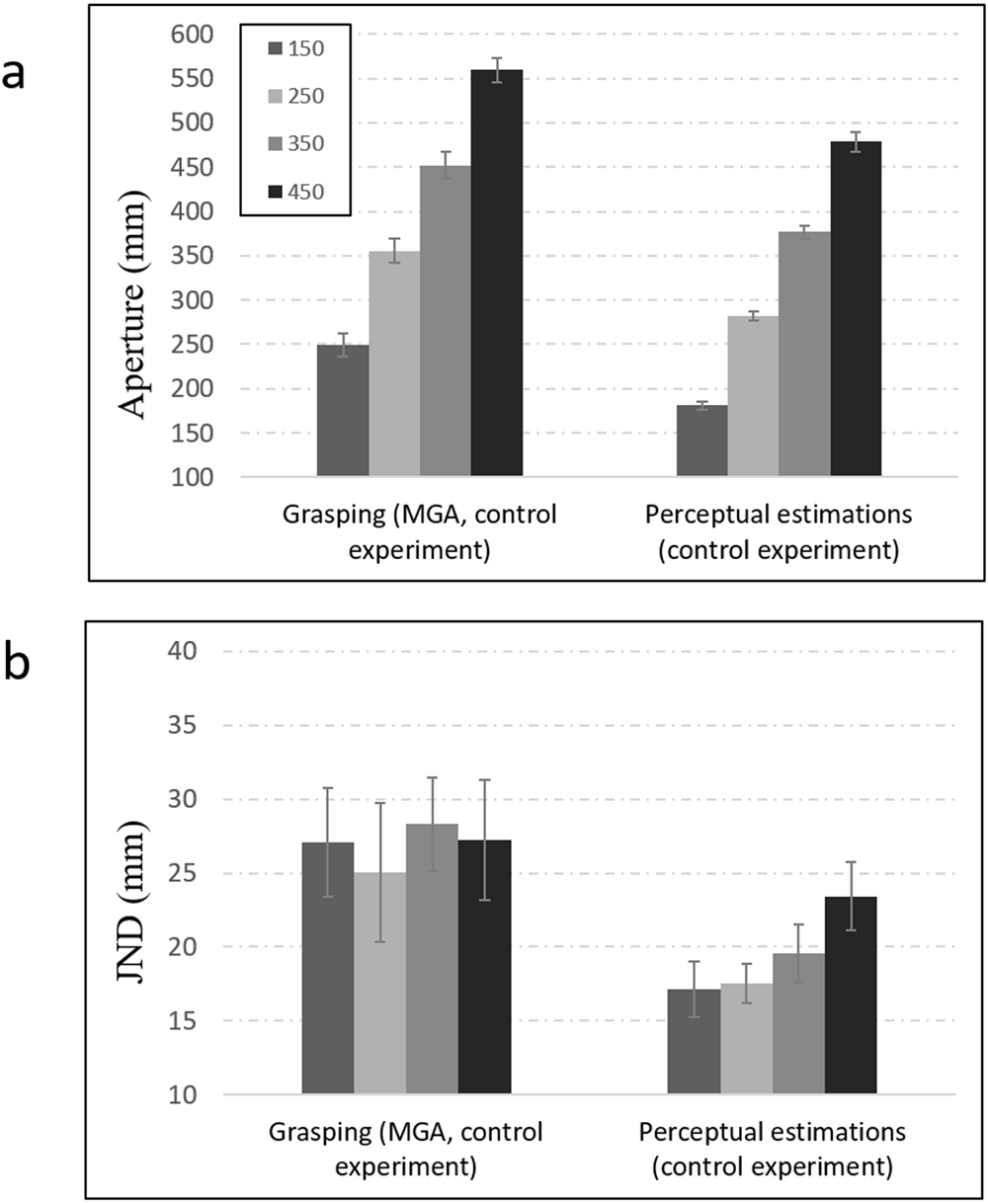
Average grip apertures (**a**) and JNDs (**b**) at the point of maximum grip aperture (MGA) in the control experiments during grasping and perceptual estimations. As in the main experiment, perceptual estimations adhered to Weber’s law whereby grasping trajectories were immune to Weber’s law. Error bars represent standard errors of the mean.

The results of the main experiment show that unlike for perceptual estimations, bimanual grasping movements escape the influence of Weber’s law. To rule out the possibility that the grasping movements violated Weber’s law due to online corrections based on visual feedback from the fingers and the target object^21^, we ran a control experiment in which grasping movements were performed in an open-loop fashion^22^. Participants were asked to perform bimanual grasping movements under similar conditions used in the grasping condition. Unlike as in the closed-loop experiment, participants’ vision was now occluded at the moment they have initiated their movements. Figure 4 presents the average apertures at the point in which MGAs were achieved, as well as the JNDs during that point in time. As can be seen in the figure, there was again no correspondence between JND and object size during bimanual grasping (F(1,7) <1, for the linear component of size). This finding rules out the possibility that grasping movements violated Weber’s law due to online feedback during the movement.

### Controlling for absence of tactile feedback during perceptual estimations

To rule out the possibility that the dissociation between grasping and perceptual estimations steamed from the fact that tactile feedback was provided for bimanual grasping but not for perceptual estimations, we ran a second control experiment in which participants were asked to perform bimanual perceptual estimations with tactile feedback provided at the end of each trial. The design was similar to the design of the main bimanual perceptual experiment with two differences: First, following each perceptual estimations trial, participants were asked to pick up the target object, which allowed similar tactile feedback as in the grasping condition. In addition, unlike as in the main perceptual experiment, for which the estimations were made rightward to the participant’s and the object’s midline, perceptual estimations were made above the initial position of the two hands. As can be seen in Fig. 4b, and unlike as in grasping, perceptual estimations again adhered to Weber’s law (F(1,7) = 9.63, p < 0.05, for the linear component of size). These findings show that the lack of tactile feedback in the main perceptual experiment did not account for the observed dissociation between grasping and perceptual estimations.

## General Discussion

The results of the current study reveal a marked difference between the way object size is computed for perception and for visuomotor control. As expected, perceptual resolution decreased with object size in adherence to Weber’s law. In sharp contrast to perceptual estimations of size, the resolution of the hands during bimanual grasping did not increase with object size, therefore violating Weber’s law. This dissociation between perception and action was evident throughout the entire movement trajectories^10^.

The findings extend previous research that looked at the influence of object size on grasping resolution along several aspects: First, previous studies have focused only on unimanual, precision grasps. Here, we looked at the influence of Weber’s law in a different motor task, that of bimanual grasping. Although unimanual and bimanual grasping may be mediated by shared functional and neural mechanisms^18, 23, 24^, bimanual coordination requires larger degree of cooperation between the two hemispheres and is thought to be based on different aspects of the visual scene compared to unimanual grasping^25, 26^.

Reflecting the fact that people typically use both their hands to grasp large objects that are beyond the limit size of their unimanual grasps, the objects used in the current study were considerably larger in size compared to the objects used in previous studies^7, 10–12, 14, 16, 27–29^. Therefore, the current findings do not only extend the dissociation between perception and action to a new visuomotor task, but also for a considerably larger range of object sizes, which was previously restricted by the limited finger span inherent to  unimanual grasps^16^.

The present results also help to reconcile a recent debate related to the possible contribution of biomechanical constraints during unimanual grasps. In particular, it has been argued that when people grasp relatively large objects, the standard deviation of the finger aperture during precision grasps may not provide a sensitive measure for the underlying visual resolution. Under such situations, biomechanical limitations of the maximum finger span could limit the deviation of the response^15^. Consider, for example, an experimental apparatus in which the target objects are considerably large, beyond the span limit of the fingers. Obviously, under such an experimental design, the deviation of the response will not increase with object size. It is important, therefore for researchers in the field to be conscious as to the obvious limitation of finger span when designing their experiments. Such limitation is likely to affect the results when either very large objects (relative to the aperture span of the task in hand) are presented^17^ or when subjects tend to increase their aperture safety margin due to visual uncertainties inherent to the experimental design^15^. Fortunately, most previous studies that looked at the standard deviations during precision grasping were conscious to this issue and used objects of reasonable sizes, within the comfort range of unimanual grasp^7, 10–14, 16, 27–29^. Furthermore, two recent studies that directly tackled the issue of biomechanical constrains during pantomimed grasping and during manual estimations by considering individual differences in hand-size showed that when the potential hazard of biomechanical limit is accounted for, standard deviations still provide a sensitive measure of the underlying resolution^16, 30^. The results of the current study clearly show, that in a situation in which biomechanical constrains are less likely to affect performance during a bimanual grasping task, the dissociation between perception and action in terms of their adherence to Weber’s law persists. These findings strongly suggest that the mechanisms that mediate our perception of the visual world are subserved by different computations than those that mediate visuomotor interactions with objects^5^.

## Conclusions

Our findings show that the fundamental dissociation between perception and action in terms of their adherence to Weber’s law extends to situations beyond unimanual (precision) grasps. This dissociation between perception and action has been suggested to reflect the analytic processing style inherent to visuomotor control, which is substantially different than the relative processing style which characterizes perception^1, 5, 6, 31^. It is possible, therefore, that the psychophysical aspects of different types of actions, beyond grasping, are qualitatively different than those that mediate visual perception.
